# Clinical outcomes of pediatric osteomyelitis

**DOI:** 10.1186/s12887-023-03863-z

**Published:** 2023-02-03

**Authors:** Kylie Disch, Deirdre A. Hill, Harry Snow, Walter Dehority

**Affiliations:** 1 The University of New Mexico School of Medicine, Albuquerque, USA; 2 Department of Internal Medicine, The University of New Mexico School of Medicine, Albuquerque, USA; 3 Clinical and Translational Science Center, The University of New Mexico School of Medicine, Albuquerque, USA; 4 Department of Pediatrics, Division of Infectious Diseases, The University of New Mexico School of Medicine, MSC10 5590, 1 University of New Mexico, Albuquerque, 87131-0001 USA

**Keywords:** Osteomyelitis, Fracture, Pathologic fracture

## Abstract

**Background:**

Osteomyelitis in children may produce severe sequelae. However, the frequency and distribution of such complications by type of osteomyelitis (chronic or acute) is not well described.

**Methods:**

We searched the HealthFacts® database (containing medical information on 68 million individual patients in the United States) with 238 International Classification of Diseases (ICD) version 10 codes for acute osteomyelitis and chronic osteomyelitis appearing in 2015. Outcomes were recorded for each subject, including development of limb length discrepancies, pathologic fractures, mortality, and need for multiple surgeries or prolonged orthopedic care (one to two years following diagnosis). Gender, age and season of diagnosis were also assessed. Chi-square tests were used to compare differences between categorical variables, and t-tests between continuous variables.

**Results:**

Eight hundred sixty-nine subjects were included (57.4% male). Children with chronic osteomyelitis were older than those with acute osteomyelitis (median 9.5 years vs 12.0, respectively, *p* = .0004). Diagnoses were more common in winter (*p* = .0003). Four subjects died while hospitalized during the study period (two with acute osteomyelitis, two with chronic osteomyelitis). Limb length discrepancies were rare and similarly distributed between infection types (≤ 1.3% of subjects, *p* = .83). Subjects with chronic osteomyeltis were more likely to require long-term orthopedic follow-up (14.0% vs. 4.8% for acute osteomyelitis, *p* < .0001), suffer from pathologic fractures (1.5% vs < 1.0%, *p* = .003) and to require multiple surgeries (46.0% vs. 29.3%, *p* = .04).

**Conclusions:**

Though infrequent, serious outcomes from osteomyelitis are more common with chronic osteomyelitis than acute osteomyelitis.

## Background

Osteomyelitis in children may be associated with poor outcomes and severe sequelae [1, 2]. Complications such as limb length discrepancies and pathologic fractures may occur, as well as persistent difficulties with weight bearing and limited range of motion of affected extremities [3]. Improved awareness of the frequency and types of sequelae associated with each presentation of osteomyelitis would help clinicians provide prognostic guidance and focus future research efforts towards the most relevant areas. Despite this need, scant information exists as to the frequency and types of adverse clinical outcomes associated with pediatric osteomyelitis. Existing data is primarily limited to small, single site reports or systematic reviews of the literature. To our knowledge, no large, multi-center studies have addressed these issues. Several recently published, large studies have utilized hospitalization-based databases, which, though providing useful information on hospitalization trends over time, did not include clinical outcomes, such as limb length discrepancies, frequency of readmission, pathologic fractures or need for long-term surgical follow-up [4, 5]. Large systematic reviews of the literature have been published as well [6], but are prone to publication biases, with the potential to overestimate severe sequelae and adverse outcomes.

To address these limitations, we utilized the HealthFacts® medical database to analyze cases of pediatric osteomyelitis over a one-year period. The use of such a database allows for analysis of a larger number of cases than would be possible with a single site study, and should be more representative of a variety of outcomes (both typical and extreme) than a systematic review of published cases. In addition, the retrospective nature of the data provides an opportunity to assess rare, long-term outcomes from the infections (e.g. limb-length discrepancies) that would be very difficult to capture in large numbers with a prospective study design, and uses both inpatient and outpatient follow-up data.

We hypothesized that chronic osteomyelitis would be associated with significantly longer durations of hospital stay and higher frequencies of limb length discrepancies and pathologic fractures than acute osteomyelitis.

## Methods

Acute osteomyelitis and chronic osteomyelitis were identified using 238 unique International Classification of Diseases (ICD) version 10 diagnosis codes in HealthFacts® claims data for the year 2015. Chronic recurrent multifocal osteomyelitis cases were excluded. HealthFacts® is a healthcare claims database that is constructed using data from a broad network of hospitals, clinics, and other facilities in the United States. This database includes data on over 68 million unique patients and 478 million encounters, including Medicaid and uninsured patients. Data in HealthFacts® is extracted directly from the electronic medical record from hospitals in which the Cerner corporation has a data use agreement. Encounters may include pharmacy, clinical and microbiology, admission and billing information from affiliated patient care locations. All admissions, laboratory orders and specimens are date and time stamped, providing a temporal relationship between treatment patterns and clinical information.

Children under the age of 18 years diagnosed with acute osteomyelitis or chronic osteomyelitis in 2015 were eligible for analysis. Each subject was assigned a unique subject identification number, and was counted only once (e.g. subsequent encounters may have contributed to outcomes, such as readmissions, but were not considered separate cases). Those diagnosed with subacute osteomyelitis were considered to have chronic osteomyelitis, and those with both septic arthritis and acute osteomyelitis were considered to have acute osteomyelitis for the purposes of the analysis. Season of diagnosis was quantified using the date of first visit for an osteomyelitis in the HealthFacts® dataset as follows: winter, December 21^st^ through March 20^th^, 2015; spring, March 21^st^ through June 20^th^, 2015; summer, June 21^st^ through September 20^th^, 2015; and fall, September 21^st^ through December 20^th^, 2015. Pathogens isolated and antimicrobial susceptibility results were identified using the HealthFacts® microbiology and microbiology susceptibility datasets, respectively. Site of pathogen isolation was identified from descriptions in the microbiology dataset. Given the inability of HealthFacts® to accurately differentiate sterile cultures from cultures which were not sent (both may be reported as an absence of microbiological data), calculation of a denominator and hence a percentage of cases attributable to a given pathogen could not be performed.

All complications and sequelae were assessed in the two years following the initial diagnosis. Limb length discrepancies were identified using 22 different ICD-10 diagnosis codes. Long-term orthopedic follow-up was defined as the need for orthopedic follow-up between one and two years following the initial diagnosis. Surgeries related to the osteomyelitis diagnosis were quantified by the assessment of Healthcare Common Procedure Coding System (HCPCS) procedures codes. Such surgeries could be related to acute care (e.g. drainage of an abscess) or follow-up care (e.g. placement or removal of an orthopedic implant). Fractures coded as ‘pathologic fractures’ following the diagnosis of infection were assessed, as were readmissions for complications related to the initial diagnosis. Placement of a peripherally inserted central catheter (PICC) was determined through query of 15 different Current Procedural Terminology (CPT) codes. Information on the specific bone(s) involved was not reliably available given the large number of poorly descriptive ICD-10 codes utilized for the localization of many subjects’ site of infection in the database (e.g. lower leg, foot, etc.). As the HealthFacts® database is not based upon insurance status (and hence does not assess a fixed population of a known number, as would be the case with a Medicare database, e.g.), we were unable to calculate incidence or prevalence data, given the absence of a known denominator.

Descriptive statistics, including means and quartiles, were calculated according to type of osteomyelitis. Differences for categorical variables were determined using the chi-square statistic, and t-tests for mean differences or Wilcoxon rank sum tests/two-sample median test for median differences (for non-normally distributed variables) in distributions by osteomyelitis type and for assessment of age differences. A two-tailed *p*-value of < 0.05 was considered statistically significant. All statistical analyses were conducted using Statistical Analysis Systems (SAS) software (v. 9.4, Cary, N.C) or STATA (v. 12.1, College Station, TX). The University of New Mexico Institutional Review Board determined this study was exempt.

## Results

Of 2,433 patients under the age of 18 years with an osteoarticular infection in the database during the year analyzed, 525 could not be categorized by a specific type of osteoarticular infection (acute osteomyelitis, chronic osteomyelitis or septic arthritis) due to coding irregularities from the dataset and were excluded. After excluding 1,039 children with septic arthritis alone, we were left with 869 children with osteomyelitis available for analysis.

Acute osteomyelitis was the most common presentation, representing 60.5% of all cases (Table 1; 33 subjects were diagnosed with subacute osteomyelitis). Chronic co-morbidities were present in 92 subjects (10.6%), with hematologic and oncologic disorders (30 subjects), pressure ulcers (22 subjects) and diabetes (13 subjects) most commonly reported.

**Table 1:** Clinical Characteristics of 869 Children with Osteomyelitis (Acute and Chronic)

Variable	Acute		Chronic		*p*-value
	n	%	n	%	
	526	60.5	343	39.5	
**Gender**					
Female	213	40.5	157	45.8	
Male	313	59.5	186	54.2	.12
**Race**					
African American	119	22.6	64	18.7	
Asian	12	2.3	4	1.2	
Asian/Pacific Islander	1	0.2	2	0.6	
Biracial	8	1.5	4	1.2	
Caucasian	324	61.6	233	67.9	
Hispanic	18	3.4	4	1.2	
Native American	4	0.8	10	2.9	
Other	28	5.3	15	4.4	
Unknown	12	2.4	7	2.1	.04
**Age**					
Median(IQR)	9.5	(4.0–13.0)	12.0	7.0–15.0	0.0004
**Required Orthopedic Follow-up > 1 Year and < 2 Years (%)**	25	4.8	48	14.0	< .0001
**Region**					
Northeast	33	6.3	28	8.2	
Midwest	192	36.5	145	42.3	
South	266	50.6	139	40.5	
West	35	6.7	31	9.0	.03
**Time of year** **Of Diagnosis**					
Spring	126	24.0	62	18.1	
Summer	103	19.6	66	19.2	
Fall	135	25.7	63	18.4	
Winter	162	30.8	152	44.3	.0003
**Insurance Status**					
Medicaid	171	32.5	113	32.9	
Private/HMO	154	29.3	108	31.5	
Govt/Military	34	6.5	17	5.0	
Self-pay	20	3.8	8	2.3	
Not stated	147	27.9	97	28.3	0.350
Pathogens Isolated^*^					
MSSA	51		6		
MRSA	41		13		
*S. aureus* NOS	33		10		
*Staphylococcus spp.*	29		15		
*S. pyogenes*	7		2		
*Salmonella spp.*	3		0		
*Pseudomonas spp.*	7		3		
*Serratia spp.*	1		2		
**Culture growth:**					
Blood culture	61	48.8	7	21.2	
Bodily fluid (e.g.synovial fluid, abscess)	5	4.0	3	9.1	
Wound/tissue culture (including bone/bone marrow)	55	44.0	20	60.6	
Other^a^	4	3.2	3	9.1	0.238

*HMO* health maintenance organization, *Govt*, government, *MSSA* methicillin susceptible *S. aureus*, *MRSA* methicillin resistant *S. aureus*, *NOS* not otherwise specified

^*^Percentages and *p*-values not calculated due to lack of a reliable denominator. The HealthFacts database does not reliably differentiate between sterile cultures and instances where cultures may not have been collected

^a^‘Other’ was a specific category in the database, with no additional information available on site of culture

Boys were more commonly affected for both types of osteomyelitis (499 boys, 57.4% vs. 370 girls, 42.6%), though this did not reach statistical significance. Children with chronic osteomyelitis were older than those with acute osteomyelitis (median 9.5 years vs. 12.0 years, respectively, *p* = 0.0004). Ethnic distribution differed by diagnosis, with chronic osteomyelitis being more common in Caucasian subjects and less common in African American children. Subjects with chronic osteomyelitis were also significantly more likely to require long-term orthopedic follow-up than those with acute osteomyelitis (14.0% vs 4.8%, *p* < 0.0001). Diagnoses were more common in the winter for all conditions (314 cases, vs. 198 cases in the fall, 188 cases in the spring and 169 cases in the summer, *p* = 0.0003), and more likely to be encountered in the Midwestern and Southern regions of the country than the Northeast or Western regions (*p* = 0.03).

*Staphylococcus aureus* was the most common organism isolated (Table 1). Of the 223 organisms reported on culture, 69.1% were *S. aureus*, with 35.1% of these being methicillin resistant *S. aureus* (MRSA). Of note, only 51 organisms (22.9% of all organisms in the study) were isolated on cultures from children with chronic osteomyelitis. Of all cultures with growth of a suspected pathogen, isolation of the organism on blood culture was more common in acute osteomyelitis than chronic osteomyelitis, while isolation of the organism on wound or tissue cultures was more common in chronic osteomyelitis than acute osteomyelitis, though this did not reach statistical significance.

Death was rare, and affected only four hospitalized subjects during the study period (two subjects with acute and chronic osteomyelitis each). Forty-seven subjects required intensive care.

Limb length discrepancies were also rarely encountered, and were not differentially distributed between types of osteomyelitis (1.3% of subjects with acute osteomyelitis, 1.2% of those with chronic osteomyelitis, *p* = 0.83 (Table 2)). Subjects with chronic osteomyelitis were more likely to require multiple surgeries (46.0%) than children with acute osteomyelitis (29.3%, *p* = 0.04). The need for readmission was not significantly different between infection types (5.8% for chronic osteomyelitis vs 6.7% for acute osteomyelitis, *p* = 0.31). Pathologic fractures were significantly more common in children with chronic osteomyelitis (*p* = 0.003), though were a rare occurrence, and present only in eight subjects (involving the femur in three cases, the tibia and humerus in two cases each and one unspecified bone). Pathologic fractures varied by race (*p* = 0.002), with a higher frequency in Caucasians than all other races combined, though comparisons of other clinical outcomes by race were not significant. Only 3 subjects required surgical amputation and only 2 required skin grafting. Clinical complications did not vary by insurance status.

**Table 2:** Clinical Outcomes of Osteomyelitis in Children (*n* = 869 defined by groups below)

Clinical Outcome	Acute Osteomyelitis		Chronic Osteomyelitis		*p*-value
	***N* (526)**	**%**	***N* (343)**	**%**	
**Hospital Stay (Days)**					
Median (IQR)	4.0 (2–7)		1.0 (1–5)		< .0001
**Required surgery (%)**	75	14.3	63	18.4	.11
**Required > 1 surgery (%)**	22	29.3	29	46.0	.04
**Subjects with LLD**	7	1.3	4	1.2	.83
**Duration of IV antibiotic**					
**Therapy prescribed (days)**	(*N* = 59)		(*N* = 23)		
Median (IQR)	1 (1–2)		2 (1–3)		.17
**Route of Administration:**	*n* = 306		*N* = 132		
Oral	65	21.2	31	23.5	
Intravenous	59	19.3	23	17.4	
Intramuscular	26	8.5	10	7.6	
Injectable	134	43.8	59	44.7	
Topical	20	6.5	7	5.3	
Other	2	0.7	2	1.5	.92
**Required PICC placement (%)**	36	6.8	13	3.8	.06

*Abbreviations: IQR* intraquartile range, *LLD* leg length discrepancy, *PICC* peripherally inserted central catheter

## Discussion

To our knowledge, our study is one of the largest reports of pediatric osteomyelitis compiled, and the largest including assessments of clinical outcomes, seasonality and chronic osteomyelitis. Okubo provided a description of 3,730 hospitalizations for acute osteomyelitis [4]. However, the unit of analysis for this study was the hospitalization, and individual patient-level data was not available. In addition, these studies did not assess complications and sequelae nor microbiological outcomes, nor did they include children with chronic osteomyelitis. Dartnell described a literature review of over 12,000 cases of osteoarticular infections in children [6]. However, this focused on the epidemiology, clinical presentation and microbiology of these conditions, with limited information on follow-up data and outcomes. In addition, these were published reports (and not inclusive of all cases in a clinical dataset, as in our study), and hence at high risk for publication bias, particularly as relates to unusual clinical outcomes.

Though infrequent, we found a higher burden of severe sequelae in subjects with chronic osteomyelitis, a patient population which is understudied. Children with chronic osteomyelitis were significantly more likely to experience pathologic fractures than children with acute osteomyelitis, and more likely to require multiple surgeries than those with acute osteomyelitis. Children with chronic osteomyelitis were also significantly more likely to require long-term orthopedic follow-up than children with acute osteomyelitis, though given the chronic nature of the disease, this is not surprising. Interestingly, the frequency of limb length discrepancies did not vary between groups, which was contrary to our hypothesis of a higher frequency in children with chronic osteomyelitis. Median hospital stays were higher for acute osteomyelitis, perhaps reflective of increased stability and less severe systemic illness among children with chronic osteomyelitis permitting more rapid transition to outpatient management.

Our results regarding sequelae from acute osteomyelitis were largely in line with outcomes from smaller studies. Mediamolle reported that 2.8% of 71 young infants developed a limb length discrepancy following a bone or joint infection [7], while Wang found limb length discrepancies in one of 58 children with septic arthritis (1.7%) [8]. McNeil described a retrospective, single site assessment of 286 children with acute osteoarticular infections due to *S. aureus*, in which limb length disorders were noted in 1.4% of subjects [9]. As a whole, these reports are similar to our finding of limb length discrepancies in 1.3% of children with acute osteomyelitis. Pathologic fractures were seen in 2.8% in the McNeil study [9], slightly higher than seen with our data. Readmissions in 8.7% of 195 children with osteomyelitis were reported by Vorhies [10], though only two subjects in this report had chronic osteomyelitis. This is similar to our finding of readmissions in 6.7% in subjects with acute osteomyelitis. Long-term orthopedic involvement was 4.8% for acute osteomyelitis in our study, similar to the findings of Vorhies (7.9%) [10].

*S. aureus *was the most commonly isolated organism, as previously described [6, 11]. We also documented a relative inability to isolate a pathogen in chronic osteomyelitis compared with acute osteomyelitis (only 22.9% of all organisms isolated in the dataset were from children with chronic osteomyelitis). Such difficulties with cultivation may impact selection of appropriate antimicrobial agents and potentially confound diagnosis for this condition. Given that chronic osteomyelitis may at times resemble neoplasms both clinically and radiologically [12], increased confidence in microbiology results may help distinguish between these entities. Use of metagenomic next generation sequencing or other novel diagnostic technologies may be beneficial in this setting, and is deserving of more study.

The male predominance in our dataset is similar to other reports [4, 13]. The mean age of subjects with acute osteomyelitis from our study is in keeping with previous reports as well [4, 10], though our finding that children with chronic osteomyelitis were older than children with acute osteomyelitis has not previously been reported from a large dataset. This may stem from the fact that the establishment of chronic osteomyelitis may be less reliant upon hematogenous spread, as is often seen in younger children [2].

We found that acute and chronic osteomyelitis were significantly more common in the winter than in other seasons. Interestingly, this is in contradiction to previously reported findings. Lindsay described a 2017 cohort of 209 children with acute osteomyelitis in which diagnoses were significantly more common in summer months, and least often encountered in the winter (34.9% vs. 17.7%) [14]. However, Lindsays’ cohort was from a single study site, contained a much smaller number of subjects and included only children with acute osteomyelitis, and as such may not be as generalizable. The rationale for the regional differences we noted (significantly more cases arising from the Midwestern and Southern regions) is not readily apparent, and may be due to differential reporting and/or differential presence of HealthFacts® facilities in those areas.

Our study had several limitations. The database was limited in the specific risk factors available for analysis. However, retrospective data collection facilitated the assessment of rare long-term outcomes (such as limb length discrepancy and pathologic fractures) in a large group of subjects, a task which would be difficult to achieve in a prospective study format. Individuals whose medical records are included in the HealthFacts® database may also have received care from facilities not included in the database, may have been lost to follow-up, or the diagnosis may have been delayed outside of the two-year follow-up window we used. As a result, the database may underestimate healthcare that is not part of a single continuous episode, such as long-term follow-up. However, this would most likely produce underascertainment of sequelae, implying a non-differential misclassification of outcomes according to exposure (type of osteoarticular infection), and as a result, would tend to result in diminishment of differences between groups [15]. Thus, our results are likely to be underestimates of the true relationships with outcomes in this large healthcare claims database. Unfortunately, our dataset was lacking detailed information on microbiologic and genetic testing (e.g. panton-valentine-leukocidin presence in MRSA isolates, resistance gene carriage, PCR testing for pathogens). The frequency of pathogen identification in our study was lower than in other published series, raising concerns of incomplete recording of this data and a subsequent reporting bias [10, 16, 17]. In addition, given limitations in diagnostic coding and our inability to review actual radiographs, we were unable to assess for growth plate involvement. Furthermore, longer follow-up time may have captured additional cases of limb length discrepancies. Lastly, specific details of therapy, such as dosing of antibiotics, compliance with and duration of oral therapy, etc. were unavailable, and would be better assessed in prospective studies.

## Conclusion

Complications from osteomyelitis in children are relatively rare, though appear more frequent in children with chronic osteomyelitis. Further study of chronic osteomyelitis in children is warranted.

## Data Availability

The datasets analyzed during the current study are not publicly available due to data use agreements between the University of New Mexico and HealthFacts®, but may be available from the corresponding author on reasonable request.

## References

[1] Johnston JJ, Murray-Krezan C, Dehority W (2017). Suppurative complications of acute hematogenous osteomyelitis in children. J Pediatr Orthop B.

[2] Peltola H, Paakkonen M (2014). Acute osteomyelitis in children. New Engl J Med.

[3] Brischetto A, Leung G, Marshall CS, Bowen AC (2016). A retrospective case-series of children with bone and joint infection from Northern Australia. Medicine.

[4] Okubo Y, Nochioka K, Testa M (2017). Nationwide survey of pediatric acute osteomyelitis in the USA. J Pediatr Orthop B.

[5] Gafur OA, Copley LA, Hollmig ST, Browne RH, Thornton L, Crawford SE (2008). The impact of the current epidemiology of pediatric musculoskeletal infection on evaluation and treatment guidelines. J Pediatr Orthop.

[6] Dartnell J, Ramachandran M, Katchburian M (2012). Haematogenous acute and subacute paediatric osteomyelitis. J Bone Joint Surg.

[7] Mediamolle N, Mallet C, Aupiais C (2018). Bone and joint infections in infants under three months of age. Acta Paediatr.

[8] Wang CL, Wang SM, Yang YJ, Tsai CH, Liu CC (2003). Septic arthritis in children: relationship of causative pathogens, complications, and outcome. J Microbiol Immunol Infect.

[9] McNeil JC, Vallejo JG, Kok EY, Sommer LM, Hulten KG, Kaplan SL (2019). Clinical and microbiologic variables predictive of orthopedic complications following *Staphylococcus aureus* acute hematogenous osteoarticular infections in children. Clin Infect Dis.

[10] Vorhies JS, Lindsay EA, Tareen NG, Kellum RJ, Jo CH, Copley LA (2019). Severity adjusted risk of long-term adverse sequelae among children with osteomyelitis. Pediatr Infect Dis J.

[11] Goergens E, McEvoy A, Watson M, Barrett I (2005). Acute osteomyelitis and septic arthritis in children. J Paediatr Child Health.

[12] Pugmire BS, Shailam R, Gee MS (2014). Role of MRI in the diagnosis and treatment of osteomyelitis in pediatric patients. World J Radiol.

[13] Kremers HM, Nwojo ME, Ransom JE, Wood-Wentz CM, Melton LJI, Huddleston PMI (2015). Trends in the epidemiology of osteomyelitis. J Bone Joint Surg.

[14] Lindsay EA, Tareen N, Jo CH, Copley LA (2018). Seasonal variation and weather changes related to the occurrence and severity of acute hematogenous osteomyelitis in children. J Pediatr Infect Dis Soc.

[15] Rothman KJ, Greenland S (1988). Modern Epidemiology.

[16] McNeil JC, Forbes AR, Vallejo JG (2016). Role of operative or interventional radiology-guided cultures for osteomyelitis. Pediatrics.

[17] Arnold SR, Elias D, Buckingham SC (2006). Changing Patterns of Acute Haematogenous Osteomyelitis and Septic Arthritis. J Pediatr Orthop.

